# High fatigue scores in patients with idiopathic inflammatory myopathies: a multigroup comparative study from the COVAD e-survey

**DOI:** 10.1007/s00296-023-05344-z

**Published:** 2023-06-14

**Authors:** Silvia Grignaschi, Minchul Kim, Giovanni Zanframundo, Naveen Ravichandran, James B. Lilleker, Parikshit Sen, Mrudula Joshi, Vishwesh Agarwal, Sinan Kardes, Jessica Day, Ashima Makol, Marcin Milchert, Tamer Gheita, Babur Salim, Tsvetelina Velikova, Abraham Edgar Gracia-Ramos, Ioannis Parodis, Elena Nikiphorou, Tulika Chatterjee, Ai Lyn Tan, Miguel A. Saavedra, Samuel Katsuyuki Shinjo, Nelly Ziade, Johannes Knitza, Masataka Kuwana, Arvind Nune, Oliver Distler, Hector Chinoy, Lorenzo Cavagna, Vikas Agarwal, Rohit Aggarwal, Latika Gupta, COVAD study group, Bhupen Barman, Yogesh Preet Singh, Rajiv Ranjan, Avinash Jain, Sapan C Pandya, Rakesh Kumar Pilania, Aman Sharma, M Manesh Manoj, Vikas Gupta, Chengappa G Kavadichanda, Pradeepta Sekhar Patro, Sajal Ajmani, Sanat Phatak, Rudra Prosad Goswami, Abhra Chandra Chowdhury, Ashish Jacob Mathew, Padnamabha Shenoy, Ajay Asranna, Keerthi Talari Bommakanti, Anuj Shukla, Arunkumar R Pande, Kunal Chandwar, Döndü Üsküdar Cansu, John D Pauling, Chris Wincup, Nicoletta Del Papa, Gianluca Sambataro, Atzeni Fabiola, Marcello Govoni, Simone Parisi, Elena Bartoloni Bocci, Gian Domenico Sebastiani, Enrico Fusaro, Marco Sebastiani, Luca Quartuccio, Franco Franceschini, Pier Paolo Sainaghi, Giovanni Orsolini, Rossella De Angelis, Maria Giovanna Danielli, Vincenzo Venerito, Lisa S Traboco, Suryo Anggoro Kusumo Wibowo, Jorge Rojas Serrano, Ignacio García-De La Torre, Erick Adrian Zamora Tehozol, Jesús Loarce-Martos, Sergio Prieto-González, Raquel Aranega Gonzalez, Akira Yoshida, Ran Nakashima, Shinji Sato, Naoki Kimura, Yuko Kaneko, Stylianos Tomaras, Margarita Aleksandrovna Gromova, Or Aharonov, Ihsane Hmamouchi, Leonardo Santos Hoff, Margherita Giannini, François Maurier, Julien Campagne, Alain Meyer, Melinda Nagy-Vincze, Daman Langguth, Vidya Limaye, Merrilee Needham, Nilesh Srivastav, Marie Hudson, Océane Landon-Cardinal, Syahrul Sazliyana Shaharir, Wilmer Gerardo Rojas Zuleta, José António Pereira Silva, João Eurico Fonseca

**Affiliations:** 1 Department of Internal Medicine and Medical Therapeutics, University of Pavia, Pavia, Lombardia Italy; 2 Rheumatology Division, IRCCS Policlinico San Matteo Foundation, Pavia, Lombardia Italy; 3 Center for Outcomes Research, Department of Internal Medicine, University of Illinois College of Medicine Peoria, Peoria, IL USA; 4 Department of Clinical Immunology and Rheumatology, Sanjay Gandhi Postgraduate Institute of Medical Sciences, Lucknow, India; 5 Centre for Musculoskeletal Research, Division of Musculoskeletal and Dermatological Sciences, School of Biological Sciences, Faculty of Biology, Medicine and Health, Manchester Academic Health Science Centre, The University of Manchester, Manchester, UK; 6 Neurology, Manchester Centre for Clinical Neurosciences, Northern Care Alliance NHS Foundation Trust, Salford, UK; 7 Maulana Azad Medical College, 2-Bahadurshah Zafar Marg, New Delhi, 110002 India; 8 Byramjee Jeejeebhoy Government Medical College and Sassoon General Hospitals, Pune, India; 9 Mahatma Gandhi Mission Medical College, Navi Mumbai, Maharashtra India; 10 Department of Medical Ecology and Hydroclimatology, Istanbul Faculty of Medicine, Istanbul University, Capa-Fatih, 34093 Istanbul, Turkey; 11 Department of Rheumatology, Royal Melbourne Hospital, Parkville, VIC 3050 Australia; 12 Walter and Eliza Hall Institute of Medical Research, Parkville, VIC 3052 Australia; 13 Department of Medical Biology, University of Melbourne, Parkville, VIC 3052 Australia; 14 Division of Rheumatology, Mayo Clinic, Rochester, MN USA; 15 Department of Internal Medicine, Rheumatology, Diabetology, Geriatrics and Clinical Immunology, Pomeranian Medical University in Szczecin, Ul Unii Lubelskiej 1, 71-252 Szczecin, Poland; 16 Rheumatology Department, Kasr Al Ainy School of Medicine, Cairo University, Cairo, Egypt; 17 Rheumatology Department, Fauji Foundation Hospital, Rawalpindi, Pakistan; 18 Medical Faculty, Sofia University St. Kliment Ohridski, 1 Kozyak Str., 1407 Sofia, Bulgaria; 19 Department of Internal Medicine, General Hospital, National Medical Center “La Raza”, Instituto Mexicano del Seguro Social, Av. Jacaranda S/N, Col. La Raza, Del. Azcapotzalco, C.P. 02990 Mexico City, Mexico; 20 Division of Rheumatology, Department of Medicine Solna, Karolinska Institutet and Karolinska University Hospital, Stockholm, Sweden; 21 Department of Rheumatology, Faculty of Medicine and Health, Örebro University, Örebro, Sweden; 22 Centre for Rheumatic Diseases, King’s College Hospital, London, UK; 23 Rheumatology Department, King’s College Hospital, London, UK; 24 NIHR Leeds Biomedical Research Centre, Leeds Teaching Hospitals Trust, Leeds, UK; 25 Leeds Institute of Rheumatic and Musculoskeletal Medicine, University of Leeds, Leeds, UK; 26 Departamento de Reumatología Hospital de Especialidades Dr. Antonio Fraga Mouret, Centro Médico Nacional La Raza, IMSS, Mexico City, Mexico; 27 Division of Rheumatology, Faculdade de Medicina FMUSP, Universidade de Sao Paulo, Sao Paulo, SP Brazil; 28 Rheumatology Department, Saint-Joseph University, Beirut, Lebanon; 29 Rheumatology Department, Hotel-Dieu de France Hospital, Beirut, Lebanon; 30 Medizinische Klinik 3-Rheumatologie Und Immunologie, Universitätsklinikum Erlangen, Friedrich-Alexander-Universität Erlangen-Nürnberg, Ulmenweg 18, 91054 Erlangen, Germany; 31 Department of Allergy and Rheumatology, Nippon Medical School Graduate School of Medicine, 1-1-5 Sendagi, Bunkyo-Ku, Tokyo, 113-8602 Japan; 32 Southport and Ormskirk Hospital NHS Trust, Southport, PR8 6PN UK; 33 Department of Rheumatology, University Hospital Zurich, University of Zurich, Zurich, Switzerland; 34 National Institute for Health Research Manchester Biomedical Research Centre, Manchester University NHS Foundation Trust, The University of Manchester, Manchester, UK; 35 Department of Rheumatology, Salford Royal Hospital, Northern Care Alliance NHS Foundation Trust, Salford, UK; 36 Rheumatology Unit, Dipartimento Di Medicine Interna E Terapia Medica, Università Degli Studi Di Pavia, Pavia, Lombardy Italy; 37 Division of Rheumatology and Clinical Immunology, University of Pittsburgh School of Medicine, Pittsburgh, PA USA; 38 Department of Rheumatology, Royal Wolverhampton Hospitals NHS Trust, Wolverhampton, WV10 0QP UK; 39 City Hospital, Sandwell and West Birmingham Hospitals NHS Trust, Birmingham, UK

**Keywords:** Autoimmune diseases, COVID-19, Fatigue, Myositis, Surveys and questionnaires

## Abstract

Idiopathic inflammatory myopathies (IIMs) confer a significant risk of disability and poor quality of life, though fatigue, an important contributing factor, remains under-reported in these individuals. We aimed to compare and analyze differences in visual analog scale (VAS) scores (0–10 cm) for fatigue (VAS-F) in patients with IIMs, non-IIM systemic autoimmune diseases (SAIDs), and healthy controls (HCs). We performed a cross-sectional analysis of the data from the COVID-19 Vaccination in Autoimmune Diseases (COVAD) international patient self-reported e-survey. The COVAD survey was circulated from December 2020 to August 2021, and details including demographics, COVID-19 history, vaccination details, SAID details, global health, and functional status were collected from adult patients having received at least one COVID-19 vaccine dose. Fatigue experienced 1 week prior to survey completion was assessed using a single-item 10 cm VAS. Determinants of fatigue were analyzed in regression models. Six thousand nine hundred and eighty-eight respondents (mean age 43.8 years, 72% female; 55% White) were included in the analysis. The overall VAS-F score was 3 (IQR 1–6). Patients with IIMs had similar fatigue scores (5, IQR 3–7) to non-IIM SAIDs [5 (IQR 2–7)], but higher compared to HCs (2, IQR 1–5; *P* < 0.001), regardless of disease activity. In adjusted analysis, higher VAS-F scores were seen in females (reference female; coefficient −0.17; 95%CI −0.21 to −13; *P* < 0.001) and Caucasians (reference Caucasians; coefficient −0.22; 95%CI −0.30 to −0.14; *P* < 0.001 for Asians and coefficient −0.08; 95%CI −0.13 to 0.30; *P* = 0.003 for Hispanics) in our cohort. Our study found that patients with IIMs exhibit considerable fatigue, similar to other SAIDs and higher than healthy individuals. Women and Caucasians experience greater fatigue scores, allowing identification of stratified groups for optimized multidisciplinary care and improve outcomes such as quality of life.

**Supplementary Information:**

The online version contains supplementary material available at 10.1007/s00296-023-05344-z.

## Introduction

Idiopathic inflammatory myopathies (IIMs), a heterogenous group of rare autoimmune rheumatic diseases primarily characterized by proximal muscle weakness of limbs, with insidious to acute onset, and variable progression, is associated with significant impairment patients’ ability to perform activities of daily living [1]. This is further exacerbated by frequent extra-muscular features, including interstitial lung disease, arthritis, skin rashes, and gastrointestinal tract involvement which further contribute to disability, leading to a worsening of patients’ perception of physical health, often negatively impacting independence, social and environmental relationships, and psychological status [2].

These diseases often burden with poor quality of life (QoL) and almost every patient reports fatigue as one of their major concerns because it reduces their social, physical, and work ability [3–6].

Several bodies have recommended the use of patient-reported outcome measures (PROMs) in both clinical trials and observational studies to highlight patient’s perception of their disease, with a prominent example being the Patient-Reported Outcome Measurement Information System (PROMIS) [7, 8]. Unfortunately, owing to the rare nature of IIMs and fatigue being rarely evaluated in routine clinical practice, data on self-reported fatigue in these patients are limited.

In a recent study evaluating patients’ perception of their disease, all patients with IIMs reported the presence of fatigue, which emerged as most common and prominent symptom [9]. However, since patients’ perception of fatigue is not associated with disease activity, and the objective assessment of patient-reported physical function is often time intensive, fatigue is not a parameter evaluated in routine clinical practice [9]. VAS instruments have stood the test of time as reliable instruments to measure PROMs, owing to their ease of administration, reproducibility, and universal applicability owing to their simplicity [10, 11]. VAS-F has also demonstrated good agreement with other standard scores such as Functional Assessment of Chronic Illness Therapy-Fatigue (FACIT-F) in rheumatoid arthritis, though exploring this agreement in IIMs remains an unmet need [12]. Furthermore, triangulation with validated tools of physical function may quantify the impact of fatigue on global function and even potentially quality of life.

The aim of this study is to analyze VAS scores for fatigue (VAS-F) in an international cohort of patients with IIMs and to compare their perception of fatigue with that of patients with non-IIM systemic autoimmune diseases (SAIDs) and healthy controls (HCs).

## Methods

### Study design

The COVID-19 Vaccination in Autoimmune Diseases (COVAD) study is an ongoing international, cross-sectional, multi-center, patient-self-reported electronic survey, aimed at investigating the safety of COVID-19 vaccination in patients with SAIDs. A validated e-survey, translated into 18 languages, evaluating demographics, COVID-19 infection course, vaccination status, vaccine-related adverse effects, SAID diagnosis, disease duration, disease activity and related treatment, global health and functional status, fatigue and pain, was circulated by the COVAD study group [13, 14]. The survey followed the Checklist for Reporting Results of Internet E-Surveys (CHERRIES) to report the data [15].

All adults (≥ 18 years old), both patients with SAIDs and HCs, were reached from collaborators of the COVAD study group in their clinics and were invited to fill the e-questionnaire from December 2020 to August 2021. The study was approved by the local institutional ethics committee (IEC Code: 2021-143-IP-EXP-39) and all participants consented electronically. No incentives were offered for survey completion.

### Study variables

Demographic data evaluated in this study were age, sex, ethnicity, and country of residence, other independent variables were specific subtype of SAIDs, disease activity, and physical function.

#### Fatigue

The dependent variable was the level of fatigue experienced in the week prior to survey completion. We used the fatigue VAS, a simple tool to quantify fatigue, which can be easily adopted for the setting of a routine consultation. Fatigue was assessed using a single-item 10 cm visual analog scale (VAS). Participants were asked to place a mark on a straight line fixed at the values from zero to ten, in which zero indicated no fatigue, and ten meant the maximum fatigue experienced.

#### Disease activity and functional status

Disease activity was evaluated based on (a) physician’s assessment (patient reported increase in dose or starting of immunosuppressants was taken as a surrogate marker for physician assessment), (b) patient’s perception of the disease, assessed by a specific question (“how was your disease before the vaccination”), and (c) current glucocorticoid dose (defining active a disease that needed any dose of glucocorticoid within 4 weeks prior the vaccination). We have noted moderate agreement between patient-reported outcomes and surrogate markers for physician assessment in the COVAD study (unpublished data).

General health status and ability to perform daily activities were evaluated using five-point Likert scales (excellent/very good/good/fair/poor for general health status, and completely/mostly/moderately/a little/not at all for the ability to perform daily activities). The questions “in general, how would you rate your physical health?” and “to what extent are you able to carry out every day physical activities such as walking, climbing stairs, carrying groceries, or moving a chair?” were extracted from the PROMIS 10A short form for physical function (PROMIS 10A SF) of the PROMIS Global Health instruments [16]. The remaining five questions of the PROMIS 10A SF were not relevant for subgroup analysis by functional status. The PROMIS 10A SF outcome of fatigue was not used in the present study.

### Data extraction and statistical analysis

Data for the present study were extracted in August 2021 from the e-survey database. To avoid erroneous duplicated and incomplete entries, we meticulously excluded all incomplete entries, as well as those who did not respond to the question with VAS-F. Continuous variables were reported as mean and standard deviation (SD) for normally distributed variables and as median and interquartile range (IQR) for non-normally distributed variables. The Kolmogorov–Smirnov test was used for assessment of normality. For descriptive statistics, continuous non-normal variables were analyzed with the Kruskal–Wallis’ test, while categorical variables were analyzed by Chi-square test, with the application of Bonferroni’s correction, considering IIMs as a reference group. To evaluate the association between the VAS-F scores and our population’s characteristics, negative binomial regression multivariable analysis was performed clustering country of origin and adjusting for age, sex, and ethnicity.

We additionally conducted subgroup analyses based on disease activity according to physician assessment, patient assessment, and glucocorticoid dose, and on general health status and ability to carry out routine activities. The level of significance for subgroup analysis was set at *P* < 0.05, whereas for the post hoc analyses at *P* < 0.025. The Pearson correlation coefficient (*p*) was used to determine the relationship between VAS-F scores and disease activity, glucocorticoid dose, health status of patients. Statistical analysis was conducted using STATA 16 version.

## Results

### Population characteristics

A total of 16,327 respondents participated in the survey. After excluding incomplete and potentially duplicated responses and respondents who had not reported VAS-F, 6,988 respondents were included in the analysis, of whom 1,057 (15%) had IIMs, 1,950 (28%) had non-IIM SAIDs, and 3,981 (57%) were HCs.

The mean age of the respondents was 43.8 years (SD 16.2), with patients with IIMs older than other non-IIM SAIDs, and HCs (mean age 59.2, 49.2, 37.1, respectively; *P* < 0.001). The cohort consisted of the 72% female respondents (73.4%, 85.3%, and 65.1%, respectively; *P* < 0.001), were 55.1% White, 24.6% Asian, and 13.8% Hispanic (*P* < 0.001) (Table 1).

**Table 1:** Demographic characteristics and self-reported fatigue of the present cohort study

Variables	All	IIMs	SAIDs	HCs	*P*
	(*n* = 6988)	(*n* = 1057)	(*n* = 1950)	(*n* = 3981)	value
Age (years)	43.8 (16.2)	59.2 (14.1)	49.2 (14.0)	37.1 (13.7)	< 0.001
Female (%)	5031 (72.0)	776 (73.4)	1664 (85.3)	2591 (65.1)	< 0.001
Ethnicity					< 0.001
White	3853 (55.1)	879 (83.1)	1196 (61.4)	1778 (44.7)	
Asian	1716 (24.6)	73 (6.9)	480 (24.6)	1163 (29.2)	
Hispanic	968 (13.9)	49 (4.7)	174 (8.9)	745 (18.7)	
Others	451 (6.4)	56 (5.3)	100 (5.1)	295 (7.4)	
Fatigue VAS (0–10)	3 (1–6)	5 (3–7)^a^	5 (2–7)	2 (1–5)^a^	< 0.001

Data showed as mean (standard deviation), median (IQR), and frequency (%)

*IIMs* inflammatory idiopathic myopathies, *SAIDs* autoimmune diseases, *HCs* healthy controls

Letter in the same line (a) indicates a significant difference (*P* < 0.05) between the values

### Overall VAS fatigue

**Fig. 1:**
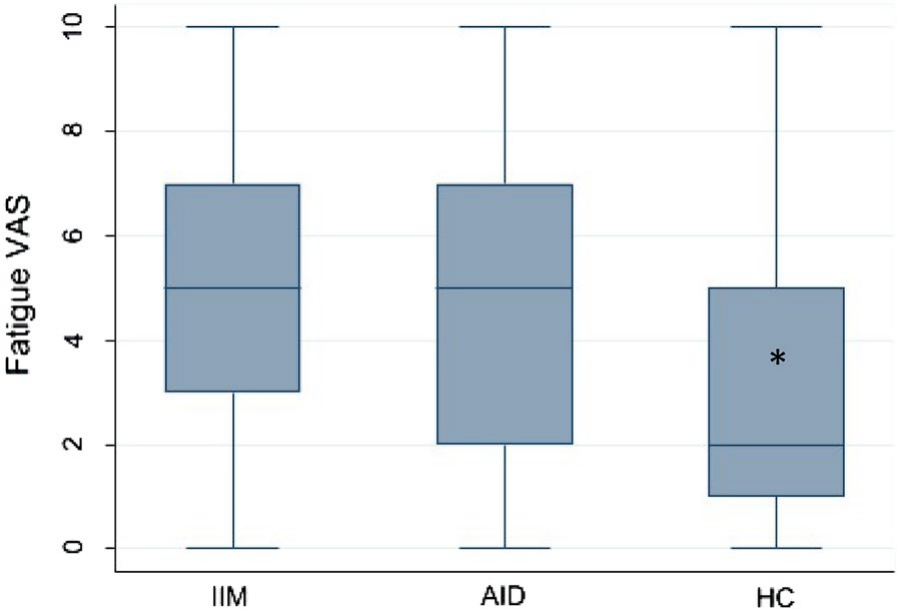
Distribution of VAS-F scores in the three-population evaluated. *IIMs* idiopathic inflammatory myopathies, *SAIDs* systemic autoimmune diseases, *HCs* healthy controls. * *P* < 0.05

The VAS-F was 3 cm (IQR 1–6) for the entire cohort. VAS-F was similar in patients with IIMs (5 cm, IQR 3–7) and non-IIM SAIDs (5 cm, IQR 2–7) (*P* = 0.084), and higher compared to HCs (2 cm, IQR 1–5; *P* < 0.001) (Table 1; Fig. 1). In multivariable analysis, VAS-F scores were positively related to female gender (*P* < 0.001) and Caucasian (*P* = 0.001) ethnicity (Table 3).

### Impact of disease activity on VAS fatigue

VAS-F scores were higher in patients with IIMs, both with active and inactive disease according to physician assessment, compared to HCs (difference of 2.1 and 1.6 cm, respectively, *P* < 0.001), but similar scores compared to non-IIM SAIDs with comparable disease activity (*P* = 0.081 and* P* = 0.052).

Conversely, when evaluating patients’ perception of disease activity, VAS-F was higher in patients with IIMs with perceived inactive disease compared to both inactive SAIDs (difference of 0.3 cm; *P* = 0.033) and HCs (difference of 1.2 cm; *P* < 0.001), whereas in perceived active disease, IIM patients’ VAS-F was significantly higher only compared to HCs (*P* < 0.001).

Based on glucocorticoid usage, both active and inactive IIMs had significantly higher VAS-F scores than HCs (*P* < 0.001) yet comparable with SAIDs (with similar disease activity) (Table 2).

**Table 2:** Multivariable binomial regression scenarios for fatigue assessed by a VAS 0–10. Models are clustered by country of origin and adjusted for age, sex, and ethnicity

Sample	Disease status	Group	Predicted VAS-F (mean)	95% CI	Difference	95% CI	*P*
All (*n* = 6.988)		IIM	4.6	4.4 to 4.8	Ref		
		AID	4.4	4.2 to 4.7	–0.2	–0.5 to 0.0	0.079
		HC	2.9	2.7 to 3.1	–1.7	–1.9 to –1.5	< 0.001
Based on physician assessment	Active + HC (*n* = 4.519)	IIM	5.0	4.7 to 5.3	Ref		
		AID	4.9	4.7 to 5.2	–0.1	–0.5 to 0.4	0.810
		HC	2.9	2.7 to 3.0	–2.1	–2.5 to –1.8	<0.001
	Inactive + HC (*n* = 6.453)	IIM	4.5	4.3 to 4.7	Ref		
		AID	4.3	4.0 to 4.5	–0.3	–0.5 to 0.0	0.052
		HC	2.9	2.7 to 3.1	–1.6	–1.8 to –1.4	< 0.001
Based on patient assessment	Active + HC (*n* = 6.361)	IIM	4.8	4.6 to 5.0	Ref		
		AID	4.7	4.5 to 4.9	–0.1	–0.4 to 0.2	0.484
		HC	2.9	2.7 to 3.1	–1.9	–2.1 to –1.7	< 0.001
	Inactive + HC (*n* = 4.879)	IIM	4.1	3.8 to 4.4	Ref		
		AID	3.8	3.6 to 4.0	–0.3	–0.6 to 0.0	0.033
		HC	2.9	2.7 to 3.0	–1.2	–1.5 to –0.9	< 0.001
Based on steroid dose	Active + HC (*n* = 4.951)	IIM	4.9	4.7 to 5.0	Ref		
		AID	4.7	4.3 to 5.0	–0.2	–0.5 to 0.1	0.232
		HC	2.9	2.7 to 3.0	–2.0	–2.2 to –1.7	< 0.001
	Inactive + HC (*n* = 6.021)	IIM	4.4	4.3 to 4.6	Ref		
		AID	4.3	4.0 to 4.5	–0.2	–0.5 to 0.1	0.275
		HC	2.9	2.7 to 3.1	–1.6	–1.8 to –1.3	< 0.001
General health status	Poor (*n* = 242)	IIM	6.8	6.5 to 7.1	Ref		
		AID	7.3	7.1 to 7.5	0.5	0.1 to 0.8	0.013
		HC	6.8	6.0 to 7.6	0.0	–0.8 to 0.7	0.957
	Fair (*n* = 1.177)	IIM	5.7	5.5 to 6.0	Ref		
		AID	5.6	5.4 to 5.8	–0.1	–0.4 to 0.2	0.412
		HC	4.3	3.8 to 4.8	–1.4	–1.9 to –0.9	< 0.001
	Good (*n* = 2.510)	IIM	4.4	4.1 to 4.6	Ref		
		AID	4.3	4.1 to 4.5	–0.1	–0.4 to 0.3	0.679
		HC	3.5	3.4 to 3.7	–0.8	–1.1 to –0.6	< 0.001
	Very good (*n* = 2.142)	IIM	3.3	2.8 to 3.8	Ref		
		AID	2.8	2.5 to 3.2	–0.5	–1.2 to 0.2	0.184
		HC	2.5	2.4 to 2.6	–0.8	–1.4 to –0.2	0.005
	Excellent (*n* = 917)	IIM	2.8	1.5 to 4.1	Ref		
		AID	1.7	1.2 to 2.2	–1.1	–2.4 to 0.1	0.079
		HC	1.8	1.6 to 2.0	–1.0	–2.3 to 0.4	0.153
Ability to carry out routine activities	Not at all (*n* = 160)	IIM	4.2	3.6 to 4.9	Ref		
		AID	4.7	3.7 to 5.6	0.4	–0.5 to 1.3	0.375
		HC	4.1	3.3 to 4.8	–0.2	–1.4 to 1.0	0.760
	A little (*n* = 482)	IIM	6.1	5.7 to 6.5	Ref		
		AID	6.2	5.8 to 6.7	0.1	–0.3 to 0.6	0.528
		HC	4.8	4.2 to 5.5	–1.3	–2.1 to –0.5	0.002
	Moderately (*n* = 964)	IIM	5.3	4.9 to 5.7	Ref		
		AID	5.7	5.4 to 5.9	0.4	–0.1 to 0.9	0.088
		HC	4.6	4.2 to 5.0	–0.7	–1.1 to –0.3	0.001
	Mostly (*n* = 1.424)	IIM	4.6	4.2 to 4.9	Ref		
		AID	4.7	4.5 to 4.9	0.1	–0.3 to 0.5	0.623
		HC	3.8	3.5 to 4.0	–0.8	–1.2 to –0.3	0.001
	Completely (*n* = 3.958)	IIM	3.2	2.9 to 3.5	Ref		
		AID	3.1	2.9 to 3.3	–0.1	–0.5 to 0.2	0.489
		HC	2.5	2.3 to 2.6	–0.8	–1.1 to –0.4	< 0.001

*VAS* visual analog scale, *CI* confidence interval, *HC* healthy controls, *IIMs* idiopathic inflammatory myopathies, *Ref* reference, *SAIDs* systemic autoimmune diseases

Regardless of the method used to assess the disease activity (physician evaluation, patients’ perception, glucocorticoid dose), after clustering by country and adjusting for age, sex, and ethnicity, in patients with both active and inactive disease, females and Caucasians reported higher VAS-F scores compared to males (reference female; coefficient −0.17; 95%CI −0.21 to −13; *P* < 0.001) and Asian (reference Caucasians; coefficient −0.22; 95%CI −0.30 to −0.14; *P* < 0.001) or Hispanic (reference Caucasians; coefficient −0.08; 95%CI −0.13 to 0.30; *P* = 0.003) ethnicities (Table 3).

**Table 3:** Multiple scenarios of binomial regression analysis of fatigue, clustered by country of origin and adjusted for age, sex, and ethnicity

			Covariates									
			Groups			Age	Male	Ethnicity				*k*
			IIMs	SAIDs	HCs			White	Asian	Hispanic	Others	
All		Coefficient	Ref.	–0.05	–0.47	0.00	–0.17	Ref.	–0.22	–0.08	–0.03	1.66
		95% CI		–0.11 to 0.01	–0.52 to –0.41	0.00 to 0.00	–0.21 to –0.13		–0.30 to –0.14	–0.13 to –0.03	–0.10 to 0.05	1.56 to 1.76
		*P*		0.082	< 0.001	0.604	< 0.001		< 0.001	0.003	0.478	< 0.001
Based on physician assessment	Active + HC	Coefficient	Ref.	–0.01	–0.55	0.00	–0.21	Ref.	–0.19	–0.09	–0.05	1.78
		95% CI		–0.10 to 0.08	–0.63 to –0.48	0.00 to 0.00	–0.25 to –0.16		–0.28 to –0.10	–0.15 to –0.03	–0.14 to 0.04	1.66 to 1.90
		*P*		0.810	< 0.001	0.441	< 0.001		< 0.001	0.005	0.279	< 0.001
	Inactive + HC	Coefficient	Ref.	–0.06	–0.44	0.00	–0.17	Ref.	–0.22	–0.07	–0.02	1.63
		95% CI		–0.12 to 0.00	–0.50 to –0.38	0.00 to 0.00	–0.21 to –0.12		–0.30 to –0.13	–0.13 to –0.02	–0.11 to 0.08	1.50 to 1.75
		*P*		0.054	< 0.001	0.806	< 0.001		< 0.001	0.010	0.723	< 0.001
Based on patient assessment	Active + HC	Coefficient	Ref.	–0.02	–0.51	0.00	–0.17	Ref.	–0.22	–0.08	–0.02	1.72
		95% CI		–0.08 to 0.04	–0.57 to –0.45	0.00 to 0.00	–0.21 to –0.12		–0.30 to –0.15	–0.13 to –0.03	–0.10 to 0.06	1.63 to 1.81
		*P*		0.484	< 0.001	0.336	< 0.001		< 0.001	0.002	0.543	< 0.001
	Inactive + HC	Coefficient	Ref.	–0.08	–0.36	0.00	–0.21	Ref.	–0.18	–0.07	–0.03	1.56
		95% CI		–0.16 to –0.01	–0.43 to –0.28	0.00 to 0.00	–0.25 to –0.17		–0.28 to –0.09	–0.13 to 0.00	–0.12 to 0.06	1.42 to 1.71
		*P*		0.030	< 0.001	0.600	< 0.001		< 0.001	0.037	0.539	< 0.001
Based on glucocorticoid dose	Active + HC	Coefficient	Ref.	–0.04	–0.52	0.00	–0.19	Ref.	–0.21	–0.08	–0.03	1.73
		95% CI		–0.11 to 0.03	–0.59 to –0.46	0.00 to 0.00	–0.23 to –0.15		–0.30 to –0.12	–0.14 to –0.02	–0.13 to 0.06	1.61 to 1.85
		*P*		0.238	< 0.001	0.602	< 0.001		< 0.001	0.015	0.475	< 0.001
	Inactive + HC	Coefficient	Ref.	–0.04	–0.43	0.00	–0.18	Ref.	–0.21	–0.09	–0.03	1.63
		95% CI		–0.11 to 0.03	–0.50 to –0.36	0.00 to 0.00	–0.23 to –0.14		–0.30 to –0.11	–0.14 to –0.03	–0.11 to 0.05	1.51 to 1.76
		*P*		0.279	< 0.001	0.540	< 0.001		< 0.001	0.002	0.418	< 0.001
General health	Poor	Coefficient	Ref.	0.07	0.00	0.00	0.00	Ref.	–0.09	0.00	–0.03	1.88
		95% CI		0.01 to 0.12	–0.11 to 0.11	0.00 to 0.00	–0.05 to 0.04		–0.19 to 0.01	–0.07 to 0.07	–0.18 to 0.11	1.75 to 2.01
		*P*		0.014	0.957	0.422	0.879		0.089	0.996	0.648	< 0.001
	Fair	Coefficient	Ref.	–0.02	–0.29	0.00	–0.07	Ref.	–0.17	–0.15	–0.01	1.96
		95% CI		–0.07 to 0.03	–0.40 to –0.17	0.00 to 0.00	–0.15 to –0.01		–0.26 to –0.08	–0.20 to –0.10	–0.07 to 0.05	1.87 to 2.05
		*P*		0.412	< 0.001	< 0.001	0.076		< 0.001	< 0.001	0.778	< 0.001
	Good	Coefficient	Ref.	–0.02	–0.22	0.00	–0.14	Ref.	–0.24	–0.03	–0.04	1.72
		95% CI		–0.09 to 0.06	–0.28 to –0.15	–0.01 to 0.00	–0.19 to –0.10		–0.33 to –0.14	–0.07 to 0.00	–0.13 to 0.04	1.58 to 1.85
		*P*		0.679	< 0.001	0.004	< 0.001		< 0.001	0.070	0.290	< 0.001
	Very good	Coefficient	Ref.	–0.16	–0.28	0.00	–0.23	Ref.	–0.23	–0.07	–0.18	1.49
		95% CI		–0.40 to 0.07	–0.45 to –0.10	–0.01 to 0.00	–0.33 to –0.14		–0.37 to –0.09	–0.14 to –0.01	–0.29 to –0.06	1.24 to 1.74
		*P*		0.177	0.002	0.010	< 0.001		0.001	0.018	0.003	< 0.001
	Excellent	Coefficient	Ref.	–0.52	–0.43	–0.01	–0.03	Ref	–0.28	–0.06	0.07	1.46
		95% CI		–1.00 to –0.04	–0.91 to 0.06	–0.02 to 0.00	–0.21 to 0.15		–0.52 to –0.04	–0.25 to 0.13	–0.40 to 0.54	0.63 to 2.29
		*P*		0.034	0.087	0.162	0.719		0.020	0.517	0.765	0.001
Routine activities	Not at all	Coefficient	Ref.	0.09	–0.04	0.00	–0.05	Ref.	–0.72	–0.41	–0.66	1.61
		95% CI		–0.11 to 0.30	–0.33 to 0.24	0.00 to 0.01	–0.30 to 0.19		–1.10 to –0.34	–0.95 to 0.14	–1.18 to –0.15	1.09 to 2.12
		*P*		0.366	0.761	0.340	0.670		< 0.001	0.141	0.012	< 0.001
	A little	Coefficient	Ref.	0.02	–0.24	0.00	–0.12	Ref.	–0.31	–0.28	–0.01	2.01
		95% CI		–0.05 to 0.09	–0.39 to –0.08	0.00 to 0.00	–0.24 to 0.01		–0.45 to –0.16	–0.40 to –0.15	–0.08 to 0.06	1.87 to 2.14
		*P*		0.527	0.003	0.085	0.068		< 0.001	< 0.001	0.754	< 0.001
	Moderately	Coefficient	Ref.	0.07	–0.14	0.00	–0.09	Ref	–0.23	0.01	–0.05	1.88
		95% CI		–0.01 to 0.16	–0.22 to –0.06	–0.01 to 0.00	–0.16 to –0.01		–0.30 to –0.15	–0.07 to 0.10	–0.14 to 0.05	1.78 to 1.99
		*P*		0.095	0.001	0.026	0.030		< 0.001	0.759	0.335	< 0.001
	Mostly	Coefficient	Ref.	0.02	–0.19	–0.01	–0.14	Ref.	–0.21	–0.01	–0.10	1.84
		95% CI		–0.07 to 0.12	–0.30 to –0.08	–0.01 to 0.00	–0.19 to –0.10		–0.29 to –0.12	–0.07 to 0.05	–0.20 to 0.01	1.68 to 2.00
		*P*		0.625	0.001	< 0.001	< 0.001		< 0.001	0.684	0.067	< 0.001
	Completely	Coefficient	Ref.	–0.04	–0.27	0.00	–0.18	Ref.	–0.27	–0.02	–0.03	1.47
		95% CI		–0.15 to 0.07	–0.38 to –0.16	–0.01 to 0.00	–0.23 to –0.13		–0.41 to 0.13	–0.08 to 0.05	–0.15 to 0.09	1.27 to 1.67
		*P*		0.486	< 0.001	0.010	< 0.001		< 0.001	0.598	0.612	< 0.001

*SAIDs* systemic autoimmune diseases, *CI* confidence interval, *HCs* healthy controls, *IIMs* idiopathic inflammatory myopathies, *k* constant, *Ref* reference

### Impact of general health on VAS fatigue

In patients who reported a “poor” perception of general health status, VAS-F scores were higher in patients with SAIDs (7.3 cm) compared to IIMs (6.8 cm). No differences were observed with HCs in the groups with “poor” and “excellent” health status. Differences between IIMs and HCs were registered in the groups with “fair”, “good” and “very good” health status (*P* < 0.005 in all status) (Table 2).

The regression analysis showed that age, sex, and ethnicity had no impact on VAS-F in patients with “poor” or “excellent” general health status. By considering Caucasian ethnicity as a reference group, lower values of VAS-F were observed in Asian patients in groups with “fair”, “good”, and “very good” health status, in Hispanic patients in groups with “fair”, and “very good” health status and in other ethnicities in the group with “very good” health status. Male sex was associated with lower levels of VAS-F in “good” and “very good” health status groups (Table 3).

### Impact of functional status on VAS fatigue

When analyzing the results according to the “ability to carry out routine activities”, VAS-F was found to be always impaired in IIMs compared to HCs (*P* < 0.005), except in the “not at all” group, while no differences were found compared to SAIDs (Table 2). Poor PROMIS PF10 scores were also associated with higher VAS-F scores (Pearson *r* = −0.477, *p* < 0.001).

Female gender was associated with higher VAS-F in the “moderately”, “mostly”, and “completely” groups, while Asian ethnicity was associated with reduced VAS-F in all groups (Table 3).

## Discussion

Our study showed that patients with IIMs had comparable VAS-F scores to non-IIM SAIDs and higher than HCs, irrespective of disease activity, global health status, and degree of functional impairment. Disease activity, as expected, was found to be related to VAS-F in patients with IIM and non-IIM SAIDs regardless of the method of measurement. Interestingly, VAS-F was lower in patients with IIMs compared to other SAIDs among those reporting “poor” general health status, suggesting that factors other than disease activity may possibly contribute to altered fatigue perception. Females and Caucasians were at greater risk of experiencing high fatigue scores, which is consistent with previous reports [17] and, for female sex, could be partially explained by the additional psychological burden of no longer having energy to manage the family business.

Chronic diseases are often associated with poor health-related quality of life (HRQoL), both when assessed by generic and specific instruments, with fatigue being important contributing factor [4, 5]. Fatigue is nearly universally reported by patients with SAIDs, and is often one of their chief concerns [6]. Nearly two-thirds of patients with SAIDs describe fatigue as profound, debilitating, and a challenge to everyday activity, leading to a reduction of their sociality, physical, and work activity [3]. Systemic inflammation and fatigue co-exist in patients with SAIDs, and there is a growing interest in deciphering the relationships between types of fatigue experienced and the immunological, cellular, and neurophysiological pathways involved [6].

We found VAS-F to be significantly higher in patients with active disease, both with IIMs and non-IIM SAIDs, compared to HCs. This may suggest a possible relationship between inflammation and fatigue. However, we also found patients with inactive disease to display higher VAS-F than HCs, raising the possibility of other contributing factors to fatigue, which could be the muscular loss due to the disease which causes hyposthenia, the extra-muscular involvement of the disease (i.e., lung, skin, joints, gastrointestinal tract), which are a priority to investigate in future studies. However, despite often being the most prominent and debilitating symptom, fatigue is not properly measured by the commonly adopted tools to evaluate disease activity in IIMs (i.e., Manual Muscle Testing-8 or serum levels of creatine phosphokinase) [9].

The strong relationship between fatigue and functional status is well known for IIMs [9, 18] as well as for almost all SAIDs [19–23]. Our results show the VAS-F in patients with IIMs is higher than HCs in almost all situations, except in the groups of patients “completely” and “not at all” able to carry out routine activities in which the excellent disease control or the severity of other conditions, respectively, is able to strongly mitigate the impact of IIMs on the functional status. Of note, the VAS-F score in patients with IIMs who are “not at all” able to carry out routine activities is lower than in all the other groups except the “completely” one. This could be explained considering the effort made by the patients in carrying out activities which would increase their fatigue. VAS-F, therefore, seems to be able to convey a great amount of information to assess health status and guide clinical management, if correctly interpreted. Indeed, it could help identifying residual disease activity, not detected by other conventional tools, or indicate the impairment between the amount of activities performed by the patient during the day and the “cost” in terms of fatigue the patient must bear. Recently, fatigue has also been found to be a major factor in reducing IIMs patients’ Work Ability Index, which can easily lead to a HRQoL reduction both though an economical and psychological burden [24]. Moreover, VAS-F is a simple tool that could be reasonably used in common clinical practice and displays moderate to strong correlation with validated tool such as PROMIS PF-20 and SF-36 PF10 [16].

The COVAD study had the primary focus of studying COVID-19 vaccination-associated adverse events. Thus, levels of fatigue were assessed in the post-vaccination period, and in many cases, following SARS-CoV-2 infection. Consequently, background levels of fatigue may have been disturbed by mental stress related to the COVID-19 pandemic, occurrence of fatigue as an adverse event following vaccination, or associated with the post-COVID-19 condition [25, 26]. Fatigue in patients with IIMs may be arising from active disease, damage due to longstanding disease, or associated comorbidities such as fibromyalgia, as well as other potential confounders. Since the COVAD study was not specifically designed to study fatigue, our study was not powered to explore the relationships between these factors and triangulate the etiology of fatigue. We hope that these aspects would be explored in future studies [27]

We fully acknowledge the limitations of recall and reporting bias associated with our study design. The absence of information on physician-reported objective measures of disease activity or damage status prevented us from correlating how patient’s personal perception of fatigue impacts their disease activity indices. Since the COVAD study was not designed specifically to study fatigue, details of the effect of comorbidities were not presently the focus of our analysis. Collection of data directly from patients can be considered both as a strength and a weakness of this study; however, given the emerging role of PROMs in patients’ self-assessment of disease activity, this remains an important area to ascertain and understand better. The online model of our survey may have led to the under-representation of low-income patients without internet access and those severely disabled [28]. However, we have tried to minimize this through the inclusion of control groups. It is also noteworthy that a significant proportion of our respondents were approached by collaborators of the COVAD study group in their clinics, which may have offset this selection bias to a certain extent.

Our study explored a very important and under-reported quality measure of life, fatigue, in the background of patients with IIMs, a rare and underrepresented disease group. A major strength of our study is the large ethnically and geographically diverse sample of patients with IIMs, which allowed effective comparisons and reduced the likelihood of type II errors, frequent when operating with a small sample size. We also stratified the results according to potential confounding factors, such as demographical and clinical variables. This is also one of the first studies to have investigated the effect of ethnicity on fatigue in patients with IIMs.

As future directions, the COVAD group aims to increase consciousness among physicians regarding the importance of assessing fatigue, to characterize patients’ reported fatigue and triangulate it with comorbidities such as fibromyalgia and mental health disorders, and other confounding factors such as COVID-19 infection, concomitant use of NSAIDs/opioids [27], and to support the implementation of scales and questionnaires to assess disease activity [16, 29].

Our study demonstrates that patients’ perception of fatigue can be assessed with an easy, reliable, and rapid tool as the VAS scale, which can be readily incorporated into clinical practice to support a more holistic evaluation of disease activity. However, assessing the concurrent validity of VAS-F with other standard scores such as FACIT-F, and the validity of minimal clinically important difference for VAS-F in IIMs, permitting longer follow-up studies remains an unmet need [12, 30]. Finally, fatigue remains an important, under-recognized, and scarcely assessed feature of SAIDs which warrants more detailed attention and study through incorporation of PROs in observation and interventional study outcome measures.

Our study found the burden of fatigue to be similar in patients with IIMs and other SAIDs, but higher than in HCs, with higher fatigue scores exhibited by females and Caucasians, and comparatively lower scores in those of Asian and Hispanic ethnicity. Physicians can easily and rapidly evaluate patients’ perception of health-related issues during outpatient clinic assessment, and our results can aid them in identifying and prioritizing patients who could be more prone to develop fatigue and who may benefit from optimized multidisciplinary care. The application of these knowledge could help in reducing or avoiding the occurrence of fatigue in SAIDs patients, thus ameliorating their quality of life and reducing socio-economic costs related to the occurrence of fatigue.

## Supplementary Information

Below is the link to the electronic supplementary material.

Supplementary file1 (DOCX 26 KB)

## Data Availability

The datasets generated and/or analyzed during the current study are not publicly available but are available from the corresponding author upon reasonable request.
